# Application of PET/CT to adjuvant chemotherapy for early lung adenocarcinoma

**DOI:** 10.1186/1749-8090-10-S1-A198

**Published:** 2015-12-16

**Authors:** Shinsuke Sasada, Yoshihiro Miyata, Takahiro Mimae, Yasuhiro Tsutani, Takeshi Mimura, Morihito Okada

**Affiliations:** 1 Department of Surgical Oncology, Hiroshima University, Hiroshima, 734-8551, Japan

## Background/Introduction

The role of adjuvant chemotherapy for stage I lung cancer is unknown. Some Japanese trials demonstrated that tegaful-uracil chemotherapy improved the prognosis of stage I lung cancer over 2 cm.

## Aims/Objectives

The purpose of this study is to determine the significance of the maximum standardized uptake value (SUVmax) on F-18-fluorodeoxyglucose positron emission tomography/computed tomography (FDG-PET/CT) images to postoperative adjuvant chemotherapy for early lung adenocarcinoma.

## Method

We reviewed 174 consecutive patients with completely resected pathological T1b-2aN0M0 lung adenocarcinoma between January 2006 and March 2011, and assessed recurrence-free interval and overall survival based on SUVmax values derived from preoperative FDG-PET/CT images. All patients were assessed by FDG-PET/CT before surgery

## Results

Ninety patients received adjuvant chemotherapy and 84 did not. Patients given adjuvant chemotherapy were older, but had the lower T status tumor than patients who were not (both, p < 0.001). Adjuvant chemotherapy conferred benefits upon recurrence-free interval and overall survival compared with observation (p = 0.007 and p = 0.004, respectively). Multivariate Cox proportional hazard analyses revealed SUVmax as an independent prognostic factor for recurrence-free interval (hazard ratio 8.03, p < 0.001). Recurrence-free interval and overall survival were significantly longer for patients who received adjuvant chemotherapy compared with those who did not in the group with SUVmax ≤ 2.6 (p < 0.001 and p < 0.001, respectively). However, recurrence-free interval and overall survival did not significantly differ between such patients in the group with SUVmax < 2.6 (p = 0.421 and p = 0.452, respectively).

## Discussion/Conclusion

Preoperative SUVmax on FDG-PET/CT images reflected the efficacy of postoperative adjuvant chemotherapy in patients with pathological T1b-2aN0M0 lung adenocarcinoma. Indications of adjuvant chemotherapy for eraly lung adenocarcinoma might be more precisely determined using SUVmax on FDG-PET/CT images together with tumor size.

